# Key Risk Factors, Sex Differences, and the Influence of High-Intensity Exercise on Colorectal Carcinogenesis: A 10-Year Cohort Study Based on 1,120,377 Individuals from the NHISS Data

**DOI:** 10.3390/curroncol31120553

**Published:** 2024-11-25

**Authors:** Hyunseok Jee

**Affiliations:** School of Kinesiology, Yeungnam University, 280, Daehak-ro, Gyeongsan 38541, Gyeongbuk, Republic of Korea; jeehs@ynu.ac.kr; Tel.: +82-53-810-3139

**Keywords:** colorectal cancer, big data analysis, exercise, exercise program development, receiver operating characteristic curve analysis

## Abstract

Colorectal cancer (CRC) is the third most common cancer globally. Therefore, this study aims to examine data from the National Health Insurance Sharing Service (NHISS) to investigate factors influencing colon cancer incidence, focusing on key variables and optimal cutoff points. The patient cohort from the NHISS database included 1,120,377 individuals aged 1–85 years. CRC data were retrieved using diagnostic codes from the Korean Standard Classification of Diseases and Causes of Death. Analyses included logistic regression and receiver operating characteristic curve assessments. In this retrospective cohort study, 1,120,377 patients were analyzed for over 10 years, including 2802 with CRC via propensity score matching (PSM). Key risk factors were blood pressure, fasting blood sugar, liver somatic index, alcohol consumption, smoking duration, and hemoglobin levels. Patients with CRC showed sex differences in gamma-glutamyl transpeptidase (GGT). High-intensity exercise (3 days/week) reduced CRC risk by 26% (*p* < 0.05). Optimal threshold points for GGT and Charlson Comorbidity Index (CCI) were 23.50 U/L (AUC, 0.52) and 1.50 (AUC, 0.58), respectively. CCI scores were higher in patients with cancer, especially men with peptic ulcers and both sexes with metastatic cancer (*p* < 0.01). Our findings reveal new risk factors and interventions, including tailored exercise programs for CRC management, highlighting the importance of enhanced preventive strategies and personalized care.

## 1. Introduction

Despite the availability of diverse treatment strategies, including postoperative medication, surgery, and alternative medicine, patients with colorectal cancer (CRC) continue to experience high mortality and morbidity rates [1,2]. The anatomical location of the colon complicates the early detection of CRC. This disease frequently metastasizes to adjacent organs and other anatomical systems, ranking it as the third deadliest and fourth most common cancer diagnosis globally, often with potentially fatal outcomes [3]. According to Statistics Korea, CRC is the third most prevalent cancer in South Korea [4]. Patients often experience complications such as anemia, cachexia, malnutrition, and depression, which can exacerbate their condition even after surgery [5].

A study shows that obesity and a sedentary lifestyle significantly increase the risk of CRC [6]. According to the American College of Sports Medicine (ACSM; www.acsm.org), exercise involves planned, repetitive, and purposeful physical activities. Exercise interventions emphasize specific prescriptions that go beyond the general health benefits of regular physical exercise. The effectiveness of these activities depends on factors such as intensity, duration, frequency, and participant demographics (e.g., age or sex). Preclinical trials using animal cancer models show that high-intensity treadmill exercise surpasses moderate-intensity treadmill exercise in efficacy, as measured based on survival rates, quality of life, and molecular analyses [7]. Exercise interventions should be tailored to specific cancer types, considering other disease-related factors [8].

The National Health Insurance Sharing Service Database (NHISS DB) is a mandatory, individual-focused tracking system that covers all South Korean citizens, operating from 2002 to 2015 [9]. Its digital format enables the tracking of medical records for the South Korean population. This comprehensive data structure supports cross-sectional, retrospective, and prospective studies. The growing body of research from the NHISS DB highlights its utility in identifying factors linked to colon cancer incidence, aiding in the development of targeted intervention programs, such as personalized exercise prescriptions [10].

Regarding the reduction of colon tumor incidence, the following hypotheses were formulated:

1 NHISS DB contains a set of significant variables that contribute to CRC incidence.
2 Sex-specific differences in key variables influence CRC risk.
3 Among these significant variables, differences in exercise modalities, such as intensity and duration, can be identified through big data analysis.
4 Optimal threshold cutoff points for these variables can be established based on the significant variables identified.
5 Charlson comorbidity index (CCI) can also influence CRC incident rates.

Therefore, this study aims to examine the NHISS DB to identify significant variables and determine optimal cutoff points for CRC patients using logistic regression analysis, providing insight for developing targeted programs to predict CRC progression and enhance symptom management.

## 2. Materials and Methods

### 2.1. Study Design

This study is designed as a retrospective cohort study utilizing the NHISS DB to investigate CRC risk factors over a 10-year period from 2009 to 2019. This is the most recent NHISS data available as of 2024. While propensity score matching (PSM) was used to construct a comparable control group, all data collection and analysis were conducted retrospectively. A total of 1,120,377 individuals were tracked, utilizing annual updates from medical records to observe changes in key health metrics over time. Furthermore, CRC-related diagnostic codes were sourced from the Korean Standard Classification of Disease and Causes of Death (KSCDCR), including C19 (malignant neoplasm of the colon with rectum), C20 (malignant neoplasm of the rectal ampulla), C218 (malignant neoplasm of the anorectum), D011 (carcinoma in situ of the rectosigmoid junction), and D012 (carcinoma in situ of the rectum), based on physicians’ diagnoses (Table S1).

All hospitals were mandated to submit medical records to the Health Insurance Review and Assessment Service (https://www.hira.or.kr/main.do). Furthermore, all cancers, including CRC, are registered with the government and reported to the WHO. The treating physician initially diagnosed CRC as the first indication of cancer. Diagnoses were confirmed and recorded based on clinical or histological findings, such as biopsies or computed tomography scans, along with risk factors. Records of subsequent recurrences, metastases, or new cancers were also maintained.

**Figure 1:**
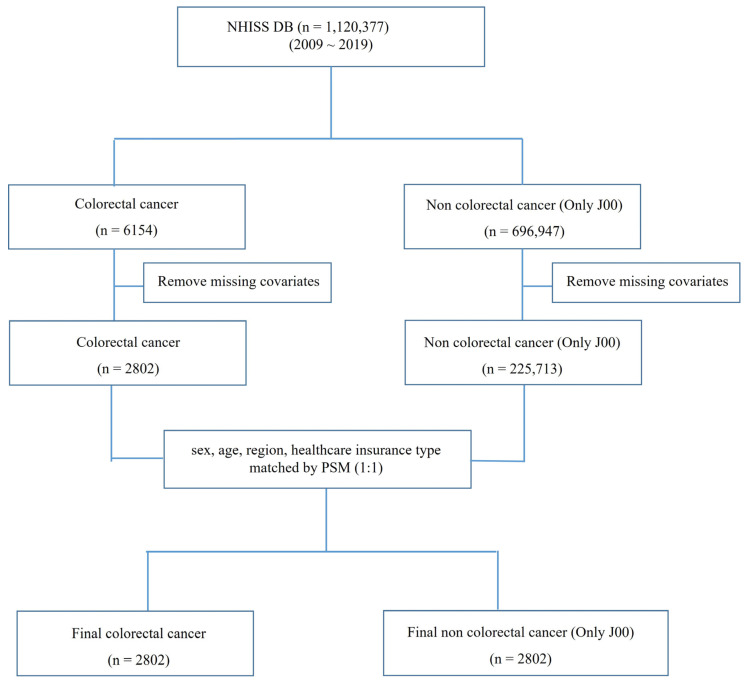
Study flowchart on data origin and analysis parameters for colorectal cancer patients. NHISS data were used in this study. From 1,120,377 registered patients, data for 6154 and 696,947 patients with colorectal cancer and common cold, respectively, were extracted. Missing covariates were removed, and sex, age, region, and type of healthcare insurance were matched using 1:1 PSM. Finally, 2802 patients—colorectal cancer and common cold—were included in the analysis. Twenty-one parameters were used for *t*-tests, logistic regression, and ROC curve analyses. NHISS DB, National Health Insurance Sharing Service Database; PSM, propensity score matching; ROC, receiver operating characteristic.

A total of 21 variables from NHISS DB were selected to identify the primary influencing factors distinguishing patients with CRC from the control group in this dataset (Figure 1). The 21 variables used were based on evidence from studies regarding their associations with CRC risk and progression, as well as data availability within the NHISS DB (Table S2). These variables encompass lifestyle factors (e.g., smoking status, alcohol consumption), physiological metrics (e.g., body mass index, blood pressure, fasting blood glucose levels), and comorbidities (e.g., Charlson Comorbidity Index), all of which have been previously linked to CRC incidence and prognosis in population studies. The selection aimed to provide a comprehensive risk profile, enabling the identification of modifiable factors that could support preventive or therapeutic strategies for CRC. For the control group, patients diagnosed with the common cold (coded J00) were identified as the least severe condition and represented the healthiest individuals registered in the NHISS DB (Table S1). This group was matched with CRC patients based on key demographic factors, such as sex, age, and region, to minimize potential confounding and facilitate a focused comparison of CRC-specific variables.

Variables associated with carcinogenesis in colorectal organs were identified. Analyzing these 21 variables across both CRC and control groups allowed for the identification of distinct factors and the establishment of optimal cutoff points that may serve as indicators for early detection and risk stratification in CRC. This approach aligned with the study’s objective to develop tailored intervention programs informed by robust, population-specific data.

These primary variables were annually tracked to assess their correlation with CRC incidence and progression and to establish optimal cutoff points.

Through this study design, NHISS data allowed for an in-depth analysis of disease progression and the temporal patterns of various risk factors in CRC patients.

### 2.2. Data Source and Subject Population

In South Korea, all citizens aged 20 and above are required to undergo medical checkups every two years and register their medical status, which facilitates the tracing of their medical histories. This system ensures regular health monitoring for the population and provides comprehensive data for health-related studies. The complete medical history of each patient from 2002 onward was digitally obtained from the NHISS (http://www.nhis.or.kr). Furthermore, samples were acquired from the NHISS DB. This open-sample cohort data, collected from nationwide surveys and hospital prescription results, was used for research purposes. The public data were surveyed thoroughly, and we finalized the dataset by applying inclusion and exclusion criteria to data from a one-million-person cohort. This study included individuals from the NHISS DB with available medical records covering a period of at least 10 years, from 2009 to 2019. To ensure data completeness and analytical consistency, patients with incomplete records or those missing data for one or more key variables were excluded (e.g., blood pressure, fasting blood glucose) over the study period. Patients were also excluded from other forms of cancer to concentrate solely on colorectal cancer. After applying the exclusion criteria outlined in Figure 1, an assessment was conducted to ensure that the final study sample remained representative of the national population. In addition, key demographic variables, including gender, age, and residence, were compared with national distributions provided by NHISS DB. This assessment confirmed that the final sample closely matched the demographic characteristics of the South Korean population, maintaining its representativeness for national-level inferences.

Individuals with nonsevere conditions (e.g., common cold) were randomly selected and free from major chronic illnesses for the control group from the NHISS DB. The NHISS DB does not focus on specific diseases but includes sample data from the population that underwent medical checks, regardless of disease presence; hence, it represents the entire population (1 million cohort). The research database comprised records for 1,120,377 patients, representing approximately 2% of the entire South Korean population, estimated at approximately 50 million individuals. These patients were randomly selected to represent the national demographic, allowing filtering using variables such as sex, age, medical insurance fees, regional location, and other stratifications.

### 2.3. Variables

This study presented the results from analyzing the most recent data available in the NHISS database, covering records till 2019.

The 21 screened variables used in this study were analyzed from 2009 to 2019 (encompassing data from 2009 to 2015 and subsequent revisions from 2016 to 2019). The variables used (along with their respective explanations) are as follows:

The variable for alcohol consumption was defined as “number of drinks per week (2009–2015)” based on the available NHISS data, which did not distinguish between types of alcohol levels of beverages. Consequently, this variable did not account for distinctions between high-alcohol and low-alcohol drinks or allow classification of heavy drinkers or binge drinkers, which is a limitation of the current study. This was updated to “number of drinks in the past year” from 2016 to 2019, when it was revised. For smoking, respondents were categorized into 1, never smoked, 2, used to smoke but quit, and 3, currently smoker. Additional variables included height (cm), weight (kg), waist circumference (cm), body mass index (kg/m^2^), high blood pressure (mmHg), low blood pressure (mmHg), and urinary protein levels (1, weakly positive; 2, positive [+1]; 3, positive [+2]; 4, positive [+3]; and 5, positive [+4]). However, to avoid multicollinearity issues, only BMI in the final model was retained due to the high correlation between BMI and both height and weight. To ensure that highly correlated variables were not included in the same model, the correlation coefficients among variables were calculated, and the variance inflation factor (VIF) was used to assess multicollinearity. Additionally, it is considered variables with a VIF greater than 10 to have a high risk of multicollinearity and excluded them from the final model.

Other health metrics assessed were hemoglobin (g/dL), fasting blood glucose (mg/dL), total cholesterol (mg/dL), triglycerides (mg/dL), high-density lipoprotein cholesterol (mg/dL), low-density lipoprotein cholesterol (mg/dL), and serum creatinine (mg/dL). serum glutamic oxaloacetic transaminase and aspartate aminotransferase (U/L), serum glutamic pyruvic transaminase and alanine aminotransaminase (SGPT; U/L), and gamma glutamyl transpeptidase (GGT; U/L). For the exercise-related questionnaires administered from 2009 to 2015, the number of times a person engaged in “≥20 min of vigorous exercise per week” or “≥30 min of moderate exercise per week” were examined. In the updated data from 2016 to 2019, the measure was defined as the number of times a person engaged in “vigorous physical activity in a week” or “moderate physical activity in a week.” The exercise-related questionnaires were obtained from NHISS DB (https://nhiss.nhis.or.kr/), which includes self-reported physical activity information collected during regular health checkups in South Korea. The NHISS DB provides data on exercise frequency, intensity, and type, enabling researchers to assess the physical activity patterns of the population.

The physical activity information within this DB has been widely used in epidemiological studies across diverse health outcomes. Although the NHISS data are based on self-reported questionnaires, previous studies have validated their reliability and correction with physical fitness and health indicators within the Korean population. These validations support the use of NHISS data as a reliable source for analyzing exercise habits in relation to colorectal cancer outcomes.

This study was approved for exemption by the Institutional Board of Seoul National University Bundang Hospital (X-1707-411-903) because all participants were anonymized, with only their ID numbers recorded to protect personal information.

### 2.4. Statistical Analyses

Data are presented as means ± standard deviation. The analysis included results from chi-square and paired *t*-test analyses. Logistic regression was employed to analyze the factors among the 21 variables associated with colorectal carcinogenesis. Logistic regression was conducted to estimate odds ratios (ORs), *p*-values, and 95% confidence intervals (CIs) for significant factors. The lack of weights in the NHISS data prevented the use of weighted logistic regression. Despite the lack of weighting, the final study was considered comparable when comparing the demographic variables to the Korean nationally representative data. Independent variables were selected for the multivariable logistic regression models based on a literature review that identified factors previously associated with colorectal cancer risk. This study aimed to include key confounders suggested by prior studies and clinical guidelines to minimize potential biases in the model, despite not employing the directed acyclic graph (DAG) theory.

The optimal cutoff points for these significant factors were determined using receiver operating characteristic (ROC) curve analysis. The optimal cutoff points for these significant factors were determined using ROC curve analysis. It should be noted that all variables used in this study were derived from validated health measures provided by the NHISS dataset. The ROC curve analysis was applied solely to identify cutoff values that optimize the sensitivity and specificity of each variable in relation to colorectal cancer risk. Statistical analyses were performed using SAS version 9.4 (SAS Institute, Cary, NC, USA) and R (version 4.3.1, http://www.r-project.org). A *p*-value of <0.05 was considered statistically significant.

## 3. Results

### 3.1. Data Characteristics

The NHISS DB contains the 10-year medical history records for 1,120,377 South Koreans, featuring detailed tracking of patients, including those with disabilities, diseases, and medical treatments. PSM was employed to match 2802 patients with CRC and non-CRC based on sex, age, region, and insurance status (Figure 1) to minimize demographic differences between the CRC patient group and the control group, thereby enabling a more accurate comparison. These scores were then used to perform a 1:1 matching, pairing CRC patients with control subjects who shared similar demographic profiles. By generating matched groups through PSM, this study effectively controlled for confounding factors, allowing for a more independent evaluation of variables associated with CRC.

The data on the 2802 patients with CRC were extracted from the NHISS DB using CRC codes defined by the KSCDCR, as detailed in the Materials and Methods section. Additionally, a matched control group of 2802 individuals with common colds was identified.

Demographics showed that high blood pressure, low blood pressure, fasting blood glucose, and GGT were significantly elevated in the CRC group (*p* < 0.05). Additionally, alcohol consumption averaged 1.04 days in the non-CRC group, compared to 1.17 days in the CRC group (*p* < 0.01). SGPT was lower in the CRC group (*p* < 0.05).

For exercise, a significant difference was observed with 20 min of high-intensity exercise. A significant difference in frequency was observed, with more individuals in the non-CRC group than in the CRC group exercising three (cancer vs. noncancer, 147 ± 5.25 vs. 200 ± 7.14) or four (cancer vs. noncancer, 74 ± 2.64 vs. 89 ± 3.18) times a week. In contrast, the CRC group had a higher number of individuals exercising once, twice, or five to seven times a week than those in the non-CRC group (*p* < 0.05). Therefore, engaging in 20 min of high-intensity exercise in moderation (three to four times a week) proved more beneficial than exercising too little or too often (Table 1).

In contrast, no statistical significance was observed for moderate exercise.

For CCI, the *p*-values for peptic ulcer, cancer, and metastatic cancer were 0.000, <0.0001, and <0.0001, respectively (Table 1).

### 3.2. Main Factors on Colorectal Carcinogenesis Between Male and Female

The analysis adjusted for variables, including GGT and CCI scores (*p* < 0.001), which ideally suppressed these variables related to colorectal carcinogenesis (Table 2).

A total of 20 major variables associated with colon carcinogenesis based on sex were identified (Table 3). Sex differences were observed in high blood pressure, low blood pressure, and GGT (higher in male patients with cancer, *p* < 0.05). In women, SGPT was lower in patients with cancer (*p* < 0.05). Additionally, a difference was observed in urine protein (all positive exhibiting higher levels in patients with cancer than in patients without it) (*p* < 0.05).

Alcohol consumption was significant only in men, with an average of 1.7 days in the CRC group and 1.49 days in the non-CRC group (*p* < 0.01).

Variables common to both sexes included significantly lower hemoglobin levels and higher blood glucose levels in the CRC group.

In CCI, the peptic ulcer was significantly different in the CRC group (654 patients) vs. the non-CRC group (565 patients) among males (*p* < 0.01). In both sexes, cancer and metastatic cancer showed significant differences, with over three times more patients in the cancer group than in the noncancer group (*p* < 0.01). The CCI scores for patients with cancer of both sexes were 2.23 ± 2.23 and 2.53 ± 2.17, respectively (*p* < 0.01) (Table 3).

### 3.3. Exercise-Related Questionnaires on Colorectal Carcinogenesis

Logistic regression analysis was employed to determine the key factors contributing to colorectal carcinogenesis. Among the examined variables, two adjusted and one high-intensive exercise variable exhibited significant differences.

Regarding exercise, the frequency of high-intensity and moderate-intensity exercise per week was examined. Among the adjusted variables, engaging in high-intensity exercise 3 days per week was significantly associated with a 26% reduction in colorectal carcinogenesis (O.R., 0.738; 95% C.I., 0.569–0.958; *p* = 0.023) (Table 4).

### 3.4. Optimal Cutoff Threshold Points for Ameliorating Colorectal Cancer Symptoms Pursued Using ROC Curve Analysis

After conducting logistic regression analysis to identify all significant factors (Table 3 and Table 4), optimal thresholds for CRC symptoms were established using ROC curve analysis (Table 5).

**Figure 2:**
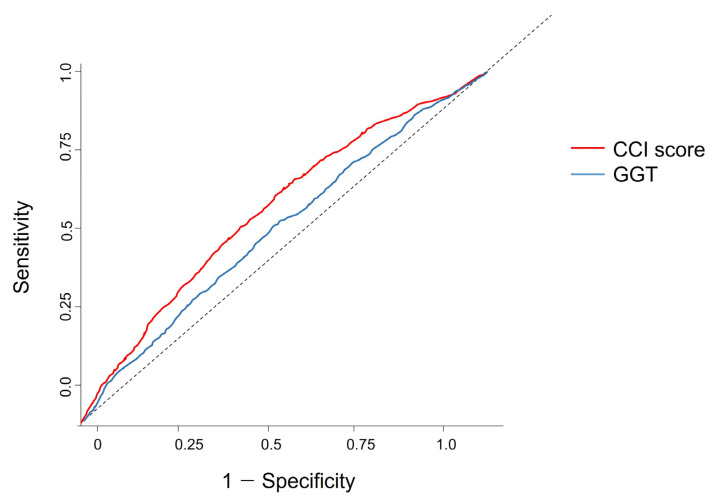
ROC curve analysis of GGT and CCI score results. The optimal threshold values for patients with colorectal cancer were 23.50 U/L for GGT (AUC 0.52) and 1.50 for CCI (AUC 0.58), with both showing statistical significance (*p* < 0.05) (Table 5). The dotted line represents the reference line, indicating a random classifier with an Area Under the Curve (AUC) of 0.5. This line serves as a baseline for evaluating the discriminatory power of our model; an AUC above this line indicates predictive accuracy better than random chance. ROC, receiver operating characteristic; CCI, Charlson comorbidity index; GGT, gamma-glutamyl transpeptidase.

Twenty-one variables were analyzed, including height, weight, waist circumference, body mass index, high blood pressure, low blood pressure, hemoglobin level, fasting blood sugar level, total cholesterol, total glyceride, high-density lipoprotein, low-density lipoprotein, serum creatinine, serum glutamic oxaloacetic transaminase and aspartate aminotransferase, SGPT, GGT, protein in urine, smoking status, alcohol consumption, exercise, and CCS score. Among these variables, GGT (0.52) and CCS score (0.58) showed high area under the curve (AUC) values. The optimal threshold points for GGT and CCS scores were 23.50 U/L and 1.50, respectively. The ROC curve analysis for CRC identified the optimal cutoff point for GGT and CCS scores (Figure 2).

## 4. Discussion

This study aimed to identify significant risk factors associated with CRC incidence and establish optimal threshold cutoff points for these variables using data from the NHISS DB. The primary research question focused on determining which demographic and clinical variables significantly contribute to CRC risk. By analyzing a large, population-based dataset, this study developed data-driven insights that could inform personalized intervention strategies to predict CRC progression and improve patient outcomes.

The following novel findings were obtained from the NHISS lifespan-based checkup database:

1 In this retrospective cohort study, the medical histories of 1,120,377 patients in South Korea were analyzed over a period exceeding 10 years (2009–2019). Among these, PSM matched 2802 patients diagnosed with CRC for analysis.
2 Several demographic and clinical risk factors for CRC were identified, including high and low blood pressure (*p* < 0.05), fasting blood sugar level (*p* < 0.01), liver somatic index (i.e., SGPT, GGT, *p* < 0.05), increased alcohol consumption (*p* < 0.01), smoking duration of 5–29 years (OR, 2.810), and hemoglobin levels (OR, 1.077).
3 Systolic blood pressure (SBP), diastolic blood pressure (DBP), GGT (higher in male patients with cancer), SGPT (lower in female patients with cancer), and urine protein (*p* = 0.046) were different (all higher in patients with cancer than those without it in the positive group). Alcohol consumption was significantly higher in men in the CRC group, who reported 1.7 days of consumption compared to 1.49 in the non-CRC group (*p* = 0.003). Common variables for both sexes included significantly lower hemoglobin levels and higher blood sugar levels in the cancer group.
4 Regarding exercise, high-intensity and moderate-intensity exercise were analyzed weekly, and among the adjusted variables, high-intensity exercise, i.e., 3 days per week, was associated with a significant 26% reduction in colorectal carcinogenesis (O.R., 0.738; 95% C.I., 0.569–0.958; *p* = 0.023) (Table 4).
5 The optimal threshold values for GGT and CCI scores were determined via ROC curve analysis as 23.50 U/L for GGT and 1.50 for CCI.
6 CCI showed a difference between the cancer (mean score: 654) and noncancer groups (mean score: 565) (*p* < 0.01) among men with peptic ulcers. Both men and women showed a significant difference in the cancer group, with more than three times as many cancer and metastatic cancer variables (*p* < 0.01). The average CCI scores for cancer patients were 2.23 ± 2.21 for men and 2.53 ± 2.17 for women, respectively (*p* < 0.01).

### 4.1. Risk Factors and Biomarkers Associated with Colorectal Cancer

Globally, mortality rates attributed to smoking, overweight, obesity, and lack of exercise were 21% (smoking) and 2% (lack of exercise), respectively [11]. Regarding past smoking history, data from medical checkups and questionnaires were used to assess smoking duration at the time of survey completion. Although smoking is generally considered causally related to CRC, the precise relationship between smoking duration and risk remains unclear [12].

Colon cancer can alter the urinary proteome, offering potential prognostic implications for its detection [13,14]. Similarly, a study shows that altered metabolic profiles are detectable through urine analysis in patients with cancer [15]; however, our findings were inconsistent. The urine protein results in this study did not show a significant difference (Table 1), possibly because such patients at risk are more vigilant about prevention. CRC is often termed “quiet cancer” owing to the absence of specific symptoms during metastasis [16], which can result in unnoticed and highly lethal metastatic symptoms.

### 4.2. Exercise Guidelines and Optimal Intensity for Colorectal Cancer Symptom Management

The American College of Sports Medicine (ACSM) and the Royal Dutch Society for Physical Therapy (KNGF) have recognized exercise as a valuable component of cancer management, with the latter publishing new guidelines for exercises in cancer management in 2022 (Dutch) and 2023 (English). However, exercise alone does not eliminate the risk of cancer.

During carcinogenesis, exercises that enhance oxidative capacity (e.g., treadmill exercise) help maintain or improve muscle mass and serve as the primary driver of physical mobility. Although resistance training stimulates muscle hypertrophy through molecular signaling pathways, it does not fully prevent muscle atrophy induced by cancer symptoms [17]. Although the ACSM recommends resistance exercise for patients with cancer, research indicates that the Akt/mTOR signaling pathway, essential for muscle growth, is unaffected or hyperactivated in cancerous conditions, differing from its regulation in healthy states [7].

Our panel data analysis revealed that engaging in 20 min of intense exercise was more effective than moderate exercise. Regarding frequency, the number of patients with cancer vs. noncancer exercising includes twice a week (227 ± 8.10 vs. 208 ± 7.42), three times a week (147 ± 5.25 vs. 200 ± 7.14), four times per week (74 ± 2.64 vs. 89 ± 3.18), five times per week (82 ± 2.93 vs. 74 ± 2.64), six times per week (46 ± 1.64 vs. 31 ± 1.11), and daily (94 ± 3.35 to 78 ± 2.78). Therefore, 3~4 times per week seems most appropriate.

High-intensity exercise thrice a week was related to a 26.2% reduction in CRC incidence (95% C.I., 0.569~0.958, *p* = 0.023) (Table 4).

This suggests incorporating high-intensity exercise into exercise programs for patients with CRC.

Preclinical trial data further confirms that high-intensity exercise is more beneficial than moderate-intensity exercise in alleviating symptoms in CRC cell lines [7]. Our findings suggest that high-intensity exercise may potentially reduce CRC incidence.

A study indicates that 10 min of intensive exercise typically mobilizes cytotoxic T and NK cells in patients with lymphoma [18]. An increase in CD8+ T cells is observed alongside a decrease in CD4+ T cell counts, indicating that immune cell responses vary based on exercise intensity. However, patients with cancer generally have a lower exercise capacity than healthy individuals. Therefore, maintaining an exercise-oriented lifestyle before a cancer diagnosis is expected to help accommodate subsequent decreases in exercise capacity and positively affect survival.

### 4.3. ROC Analysis of GGT and CCI as Indicators for Colorectal Cancer Symptoms

Through ROC and logistic regression analyses, two significant non-exercise-related variables were highlighted: GGT and CCI score (Table 5). Consequently, the cutoff values for gamma-glutamyl transpeptidase (23.50 U/L; AUC, 0.52) and CCI score (1.5; AUC, 0.58) were established as potential indicators of CRC symptoms.

Although GGT is not directly used to diagnose CRC, it is used to assess liver function in patients with cancer, and it is believed to play a crucial role in monitoring cancer progression. Elevated median GGT levels are numerically related to colorectal adenoma, and a ROC analysis involving approximately 2475 patients showed similar results (i.e., 23 IU/L) to those in this study [19].

In the CCI score, only peptic ulcer, cancer, and metastatic cancer showed significance in Table 1 and Table 2, with peptic ulcer being significant only in men (cancer vs. noncancer patients, 654 ± 37.18 vs. 565 ± 32.14, *p* < 0.01). This suggests that a CCI score of ≤1.5 for these three diseases should be avoided to reduce mortality or complications.

### 4.4. Managing Weight, Muscle Mass, and the Obesity Paradox in Cancer Patients

Maintaining an optimal weight is crucial for overall health, particularly for achieving a body composition characterized by high skeletal muscle and low-fat mass. This is particularly relevant in cancer, as muscle atrophy is linked to increased all-cause mortality and tumor recurrence in hepatocellular carcinoma [20]. Conversely, higher fat mass is related to elevated cholesterol levels, which promote carcinogenesis by deactivating relevant enzymatic interactions, ultimately increasing lipid peroxidation and enhancing carcinogenic potential [21]. This eventually leads to elevated GGT levels, a biomarker observed in this study to be significantly associated with colon cancer (*p* < 0.01; Table 3). GGT is primarily recognized as a marker for metabolic syndrome fatty liver disease, which is often used to assess problems caused by fatty liver accumulation [22]. However, in oncology, the ‘obesity paradox’ suggests a more complex relationship between body weight and cancer outcomes. While obesity is generally associated with increased cancer risk and poor outcomes, some studies indicate that overweight and mildly obese patients may have improved survival rates in certain cancers. This paradox may be due to factors such as enhanced metabolic reserves or differences in treatment tolerance, which can influence cancer prognosis. Therefore, in managing body composition for cancer patients, individualized approaches are essential, considering the potential benefits of lean muscle mass and metabolic health in optimizing survival outcomes.

In this context, myokines play a significant role in fat metabolism, particularly in the browning of white adipose tissue, which can aid metabolic health [23]. Myokines released by muscles post-exercise can enhance metabolic diseases linked to excess fat in obesity, highlighting the health benefits of an active lifestyle.

The identification of variables in this study, such as GGT and fasting blood sugar levels as novel biomarkers for colon cancer, could facilitate the development of effective and tailored exercise interventions. Individually tailored exercise programs account for various factors, including age, sex, disease status, and patient characteristics. These programs incorporate variations in intensity, duration, and frequency, with high-intensity exercise recommended in this study. These personalized exercise prescriptions are crucial as they address the diverse susceptibilities of patients with CRC, acknowledging that the effects of exercise may vary among individuals [24,25].

There is a limitation in this study, which is that PSM can introduce potential biases, especially in retrospective observational studies. While PSM helps to create a comparable control group, it does not account for unmeasured confounders, which may influence the results. To minimize bias, this study ensured balance across key covariates and conducted sensitivity analyses to assess the robustness of findings. Despite these efforts, residual confounding cannot be entirely ruled out, which represents a limitation of this approach in this study.

## 5. Conclusions

The following novel findings were obtained from the NHISS DB analysis of patients with CRC:

In this retrospective cohort study, we analyzed 1,120,377 South Korean patients over 10 years, including 2802 patients with CRC through PSM. Key risk factors identified were blood pressure, fasting blood sugar, liver somatic index (SGPT, GGT), alcohol consumption, smoking duration, and hemoglobin levels. Patients with CRC showed differences in variables such as GGT and SGPT, particularly based on sex. High-intensity exercise (3 days/week) reduced CRC risk by 26%. Optimal threshold points for GGT and CCI were 23.50 U/L and 1.50, respectively. CCI scores were significantly higher in patients with cancer, particularly among men with peptic ulcers and both sexes with cancer and metastatic cancer (*p* < 0.01).

This large-scale analysis of the NHISS DB offers valuable insights to enhance malignant symptom management in patients with CRC. Personalized high-intensity exercise interventions for patients with CRC can be effectively prescribed by considering established predictors such as weight and hemoglobin levels.

## Figures and Tables

**Table 1:** Characteristics of the NHISS DB-based study population.

Variable	All		Colorectal Cancer		Non-Colorectal Cancer		*p*-Value
	(*n* = 5604)		(*n* = 2802)		(*n* = 2802)		
	N or Mean	% or S.D.	N or Mean	% or S.D.	N or Mean	% or S.D.	
Ht	161.98	9.09	162.04	9.08	161.91	9.10	0.001
Wt	63.23	11.20	63.20	11.29	63.25	11.10	0.868
WC	83.12	8.76	83.26	8.86	82.99	8.65	0.248
BMI	24.01	3.20	23.98	3.21	24.04	3.20	0.483
SBP	127.05	15.49	127.46	15.18	126.64	15.80	0.049
DBP	77.63	9.84	78.03	10.02	77.23	9.64	0.002
Hb	13.86	1.67	13.78	1.78	13.94	1.54	0.614
FBS	105.58	30.61	107.01	33.04	104.14	27.89	0.000
TC	194.54	39.05	194.02	39.64	195.07	38.46	0.315
TG	138.86	90.68	140.20	91.72	137.52	89.62	0.270
HDL	53.68	18.96	53.67	20.10	53.69	17.74	0.967
LDL	116.02	138.61	114.15	49.61	117.89	189.64	0.313
SCr	0.96	0.69	0.95	0.65	0.96	0.72	0.737
SGOT	26.92	26.13	26.60	18.48	27.23	32.00	0.368
SGPT	24.73	22.48	24.09	21.57	25.36	23.34	0.034
GGT	41.19	60.71	43.22	65.60	39.16	55.32	0.012
**Urine Protein**							
Negative (−)	5256	93.79	2617	93.40	2639	94.18	0.296
Weakly positive (±)	157	2.80	75	2.68	82	2.93	
Positive (+1)	114	2.03	64	2.28	50	1.78	
Positive (+2)	54	0.96	32	1.14	22	0.79	
Positive (+3)	20	0.36	13	0.46	7	0.25	
Positive (+4)	3	0.05	1	0.04	2	0.07	
**Smoking status**							
Never	3131	55.87	1548	55.25	1583	56.50	0.543
Former	1317	23.50	675	24.09	642	22.91	
Current	1156	20.63	579	20.66	577	20.59	
**Alcohol consumption (1 week)**							
Average	1.10	1.77	1.17	1.83	1.04	1.71	0.005
0	3288	58.67	1604	57.24	1684	60.10	0.173
1	810	14.45	401	14.31	409	14.60	
2	546	9.74	288	10.28	258	9.21	
3	411	7.33	208	7.42	203	7.24	
4	159	2.84	89	3.18	70	2.50	
5	119	2.12	61	2.18	58	2.07	
6	79	1.41	41	1.46	38	1.36	
7	192	3.43	110	3.93	82	2.93	
**High-Intensity Exercise (1 week)**							
Average	1.00	1.77	1.01	1.81	0.99	1.73	0.629
0	3657	65.26	1832	65.38	1825	65.13	0.034
1	597	10.65	300	10.71	297	10.60	
2	435	7.76	227	8.10	208	7.42	
3	347	6.19	147	5.25	200	7.14	
4	163	2.91	74	2.64	89	3.18	
5	156	2.78	82	2.93	74	2.64	
6	77	1.37	46	1.64	31	1.11	
7	172	3.07	94	3.35	78	2.78	
**Moderate-Intensity Exercise (1 week)**							
Average	1.29	1.98	1.29	2.00	1.29	1.96	0.957
0	3294	58.78	1661	59.28	1633	58.28	0.627
1	598	10.67	295	10.53	303	10.81	
2	478	8.53	231	8.24	247	8.82	
3	435	7.76	203	7.24	232	8.28	
4	204	3.64	110	3.93	94	3.35	
5	256	4.57	124	4.43	132	4.71	
6	106	1.89	57	2.03	49	1.75	
7	233	4.16	121	4.32	112	4.00	
**CCI Comorbidities**							
Acute myocardial infarction	93	1.66	51	1.82	42	1.50	0.347
Congestive heart failure	193	3.44	99	3.53	94	3.35	0.714
Peripheral vascular accident	718	12.81	361	12.88	357	12.74	0.873
Cerebral vascular accident	723	12.90	367	13.10	356	12.71	0.661
Dementia	0	0.00	0	0.00	0	0.00	-
Pulmonary disease	2167	38.67	1067	38.08	1100	39.26	0.365
Connective tissue disorder	241	4.30	113	4.03	128	4.57	0.323
Peptic ulcer	2100	37.47	1116	39.83	984	35.12	0.000
Liver disease	294	5.25	144	5.14	150	5.35	0.719
Diabetes	1447	25.82	748	26.70	699	24.95	0.135
Diabetes complications	355	6.33	187	6.67	168	6.00	0.297
Paraplegia	49	0.87	22	0.79	27	0.96	0.473
Renal disease	119	2.12	68	2.43	51	1.82	0.115
Cancer	952	16.99	747	26.66	205	7.32	<0.0001
Metastatic cancer	146	2.61	130	4.64	16	0.57	<0.0001
Severe liver disease	33	0.59	21	0.75	12	0.43	0.116
HIV	0	0.00	0	0.00	0	0.00	-
**CCI score**	2.05	2.01	2.34	2.20	1.75	1.75	<0.0001
**CCI category**							
CCI score = 0	1349	24.07	603	21.52	746	26.62	<0.0001
1 ≤ CCI score < 3	2475	44.16	1148	40.97	1327	47.36	
3 ≤ CCI score	1780	31.76	1051	37.51	729	26.02	

The demographic characteristics of the study were provided by the NHISS DB. Data are presented as a number or mean or as a percentage or standard deviation (S.D.). NHISS DB, National Health Insurance Sharing Service database; Ht, height (cm); Wt, weight (kg); WC, waist circumference (cm); BMI, body mass index (kg/m^2^); SBP, high blood pressure (mmHg); DBP, low blood pressure (mmHg); Hb, hemoglobin level (g/dL); FBS, fasting blood sugar level (mg/dL); TC, total cholesterol (mg/dL); TG, total glyceride (mg/dL); HDL, high-density lipoprotein cholesterol (mg/dL); LDL, low-density lipoprotein cholesterol (mg/dL); SCr, serum creatinine (mg/dL); SGOT, serum glutamic oxaloacetic transaminase and aspartate aminotransferase (U/L); SGPT, serum glutamic pyruvic transaminase and alanine aminotransaminase (U/L); GGT, gamma-glutamyl transpeptidase (U/L); urine protein, protein in urine; CCI, Charlson Comorbidity Index.

**Table 2:** The analysis of the risky variables on colorectal carcinogenesis.

Variable	Unadjusted				Adjusted			
	OR	95% CI		*p*-Value	OR	95% CI		*p*-Value
**Smoking status**								
Never	1.075	0.945	1.223	0.270	1.117	0.973	1.283	0.115
Former	1.026	0.897	1.174	0.708	1.121	0.968	1.299	0.127
Current	(Ref)	(Ref)						
WC	1.004	0.998	1.010	0.248	-			
BMI	0.994	0.978	1.011	0.483	0.995	0.977	1.013	0.577
SBP	1.003	1.000	1.007	0.050	1.002	0.999	1.006	0.219
DBP	1.008	1.003	1.014	0.003	-			
**Urine Protein**								
Negative (−)	1.983	0.180	21.882	0.576	4.640	0.406	53.003	0.217
Weakly positive (±)	1.829	0.163	20.585	0.625	4.085	0.351	47.577	0.261
Positive (+1)	2.560	0.226	29.039	0.448	5.154	0.440	60.338	0.191
Positive (+2)	2.909	0.248	34.081	0.395	4.955	0.409	59.972	0.208
Positive (+3)	3.714	0.284	48.535	0.317	5.938	0.439	80.379	0.180
Positive (+4)	(Ref)	(Ref)						
Hb	0.945	0.916	0.976	0.001	0.965	0.930	1.001	0.056
FBS	1.003	1.001	1.005	0.001	1.001	0.999	1.003	0.345
TC	0.999	0.998	1.001	0.315	1.000	0.999	1.002	0.788
SGOT	0.999	0.997	1.001	0.393	1.001	0.997	1.005	0.695
SGPT	0.997	0.994	1.000	0.043	0.995	0.991	1.000	0.052
GGT	1.001	1.000	1.002	0.014	1.001	1.000	1.002	0.009
TG	1.000	1.000	1.001	0.271	1.000	1.000	1.001	0.332
HDL	1.000	0.997	1.003	0.967	1.001	0.998	1.004	0.599
LDL	1.000	0.999	1.000	0.424	1.000	0.999	1.000	0.613
SCr	0.987	0.914	1.065	0.737	0.949	0.876	1.028	0.199
**CCI score**	1.166	1.134	1.199	<0.0001	1.159	1.126	1.193	<0.0001

Data are presented as a number or mean or as a percentage or standard deviation (S.D.). WC, waist circumference (cm); BMI, body mass index (kg/m^2^); SBP, high blood pressure (mmHg); DBP, low blood pressure (mmHg); Hb, hemoglobin level (g/dL); FBS, fasting blood sugar level (mg/dL); TC, total cholesterol (mg/dL); TG, total glyceride (mg/dL); HDL, high-density lipoprotein cholesterol (mg/dL); LDL, low-density lipoprotein cholesterol (mg/dL); SCr, serum creatinine (mg/dL); SGOT, serum glutamic oxaloacetic transaminase and aspartate aminotransferase (U/L); SGPT, serum glutamic pyruvic transaminase and alanine aminotransaminase (U/L); GGT, gamma-glutamyl transpeptidase (U/L); urine protein, protein in urine; CCI, Charlson Comorbidity Index.

**Table 3:** Gender differently prevalent variables on colorectal carcinogenesis.

Variable	Male					Female				
	Colorectal Cancer		Non-Colorectal Cancer		*p*-Value	Colorectal Cancer		Non-Colorectal Cancer		*p*-Value
	N or Mean	% or S.D.	N or Mean	% or S.D.		N or Mean	% or S.D.	N or Mean	% or S.D.	
Ht	166.90	6.52	166.92	6.37	0.915	153.84	6.51	153.48	6.39	0.209
Wt	67.16	10.74	67.11	10.35	0.899	56.54	8.79	56.76	9.13	0.575
WC	85.12	8.51	84.82	7.80	0.278	80.12	8.53	79.90	9.13	0.572
BMI	24.05	3.18	24.02	3.01	0.782	23.87	3.26	24.08	3.50	0.156
SBP	128.30	14.59	127.22	15.00	0.031	126.04	16.03	125.67	17.02	0.611
DBP	78.85	9.91	77.80	9.34	0.001	76.65	10.06	76.27	10.06	0.395
Hb	14.40	1.69	14.55	1.40	0.005	12.74	1.41	12.91	1.18	0.003
FBS	108.82	34.62	105.66	29.23	0.004	103.97	29.96	101.58	25.28	0.049
TC	189.85	40.11	191.67	36.89	0.164	201.05	37.84	200.80	40.34	0.886
TG	148.18	101.43	142.92	96.40	0.116	126.74	70.48	128.43	76.05	0.600
HDL	51.58	16.92	52.11	19.31	0.387	57.20	24.16	56.35	14.34	0.333
LDL	110.40	50.21	111.83	33.80	0.322	120.47	47.95	128.09	307.40	0.429
SCr	1.03	0.67	1.04	0.80	0.618	0.82	0.59	0.82	0.55	0.871
SGOT	27.82	21.05	28.51	39.38	0.519	24.54	12.79	25.08	11.39	0.316
SGPT	26.21	25.06	27.47	26.49	0.148	20.51	13.04	21.81	16.14	0.044
GGT	53.84	77.01	48.23	66.12	0.021	25.31	32.40	23.87	21.97	0.238
Urine Protein										
Negative (**−**)	1648	93.69	1643	93.46	0.400	969	92.91	996	95.40	0.046
Weakly positive (±)	44	2.50	54	3.07		31	2.97	28	2.68	
Positive (+1)	35	1.99	37	2.10		29	2.78	13	1.25	
Positive (+2)	25	1.42	16	0.91		7	0.67	6	0.57	
Positive (+3)	7	0.40	6	0.34		6	0.58	1	0.10	
Positive (+4)	0	0.00	2	0.11		1	0.10	0	0.00	
**Smoking status**										
Never	566	32.18	591	33.62	0.388	982	94.15	992	95.02	0.114
Former	653	37.12	614	34.93		22	2.11	28	2.68	
Current	540	30.70	553	31.46		39	3.74	24	2.30	
**Alcohol consumption (1 week)**										
Average	1.70	2.06	1.49	1.92	0.003	0.28	0.80	0.27	0.84	0.702
0	735	41.79	794	45.16	0.140	869	83.32	890	85.25	0.072
1	297	16.88	314	17.86		104	9.97	95	9.10	
2	239	13.59	232	13.20		49	4.70	26	2.49	
3	200	11.37	184	10.47		8	0.77	19	1.82	
4	84	4.78	65	3.70		5	0.48	5	0.48	
5	58	3.30	55	3.13		3	0.29	3	0.29	
6	39	2.22	37	2.10		2	0.19	1	0.10	
7	107	6.08	77	4.38		3	0.29	5	0.48	
**High-Intensity Exercise (1 week)**										
Average	1.15	1.89	1.15	1.85	0.985	0.77	1.63	0.71	1.47	0.353
0	1060	60.26	1052	59.84	0.303	772	74.02	773	74.04	0.222
1	230	13.08	223	12.68		70	6.71	74	7.09	
2	154	8.75	142	8.08		73	7.00	66	6.32	
3	101	5.74	133	7.57		46	4.41	67	6.42	
4	54	3.07	68	3.87		20	1.92	21	2.01	
5	58	3.30	52	2.96		24	2.30	22	2.11	
6	32	1.82	24	1.37		14	1.34	7	0.67	
7	70	3.98	64	3.64		24	2.30	14	1.34	
**Moderate-Intensity Exercise (1 week)**										
Average	1.37	2.04	1.37	1.97	0.962	1.16	1.92	1.15	1.94	0.981
0	998	56.74	958	54.49	0.593	663	63.57	675	64.66	0.453
1	202	11.48	225	12.80		93	8.92	78	7.47	
2	151	8.58	164	9.33		80	7.67	83	7.95	
3	132	7.50	154	8.76		71	6.81	78	7.47	
4	72	4.09	70	3.98		38	3.64	24	2.30	
5	85	4.83	84	4.78		39	3.74	48	4.60	
6	36	2.05	33	1.88		21	2.01	16	1.53	
7	83	4.72	70	3.98		38	3.64	42	4.02	
**CCI Comorbidities**										
Acute myocardial infarction	39	2.22	33	1.88	0.477	12	1.15	9	0.86	0.509
Congestive heart failure	58	3.30	57	3.24	0.927	41	3.93	37	3.54	0.641
Peripheral vascular accident	203	11.54	207	11.77	0.829	158	15.15	150	14.37	0.615
Cerebral vascular accident	230	13.08	208	11.83	0.264	137	13.14	148	14.18	0.489
Dementia	0	0.00	0	0.00	-	0	0.00	0	0.00	-
Pulmonary disease	613	34.85	667	37.94	0.057	454	43.53	433	41.48	0.343
Connective tissue disorder	56	3.18	55	3.13	0.926	57	5.47	73	6.99	0.149
Peptic ulcer	654	37.18	565	32.14	0.002	462	44.30	419	40.13	0.054
Liver disease	102	5.80	98	5.57	0.774	42	4.03	52	4.98	0.293
Diabetes	464	26.38	433	24.63	0.234	284	27.23	266	25.48	0.364
Diabetes complications	130	7.39	105	5.97	0.092	57	5.47	63	6.03	0.576
Paraplegia	18	1.02	21	1.19	0.628	4	0.38	6	0.57	0.527
Renal disease	43	2.44	35	1.99	0.361	25	2.40	16	1.53	0.155
Cancer	447	25.41	125	7.11	<0.0001	300	28.76	80	7.66	<0.0001
Metastatic cancer	68	3.87	11	0.63	<0.0001	62	5.94	5	0.48	<0.0001
Severe liver disease	10	0.57	6	0.34	0.317	11	1.05	6	0.57	0.223
HIV	0	0.00	0	0.00	-	0	0.00	0	0.00	-
**CCI score**	2.23	2.21	1.68	1.76	<0.0001	2.53	2.17	1.87	1.74	<0.0001
**CCI category**										
CCI score = 0	425	24.16	504	28.67	<0.0001	178	17.07	242	23.18	<0.0001
1 ≤ CCI score < 3	708	40.25	826	46.99		440	42.19	501	47.99	
3 ≤ CCI score	626	35.59	428	24.35		425	40.75	301	28.83	

Data are presented as a number or mean or as a percentage or standard deviation (S.D.). NHISS DB, National Health Insurance Sharing Service database; Ht, height (cm); Wt, weight (kg); WC, waist circumference (cm); BMI, body mass index (kg/m^2^); SBP, high blood pressure (mmHg); DBP, low blood pressure (mmHg); Hb, hemoglobin level (g/dL); FBS, fasting blood sugar level (mg/dL); TC, total cholesterol (mg/dL); TG, total glyceride (mg/dL); HDL, high-density lipoprotein cholesterol (mg/dL); LDL, low-density lipoprotein cholesterol (mg/dL); SCr, serum creatinine (mg/dL); SGOT, serum glutamic oxaloacetic transaminase and aspartate aminotransferase (U/L); SGPT, serum glutamic pyruvic transaminase and alanine aminotransaminase (U/L); GGT, gamma-glutamyl transpeptidase (U/L); urine protein, protein in urine; CCI, Charlson Comorbidity Index.

**Table 4:** Logistic regression analysis of exercise modalities on colorectal carcinogenesis.

Variable	Unadjusted				Adjusted			
	OR	95% CI		*p*-Value	OR	95% CI		*p*-Value
High-Intensity Exercise (1 week)								
0	(Ref)				(Ref)			
1	1.006	0.846	1.196	0.944	1.053	0.853	1.299	0.633
2	1.087	0.891	1.327	0.410	1.159	0.917	1.465	0.218
3	0.732	0.586	0.915	0.006	0.738	0.569	0.958	0.023
4	0.828	0.604	1.135	0.241	0.724	0.505	1.038	0.079
5	1.104	0.801	1.522	0.546	1.187	0.821	1.715	0.362
6	1.478	0.933	2.342	0.096	1.507	0.889	2.556	0.128
7	1.201	0.883	1.632	0.244	1.223	0.853	1.753	0.274
**Moderate-Intensity Exercise** **(1 week)**								
0	(Ref)				(Ref)			
1	0.957	0.804	1.139	0.623	0.927	0.751	1.145	0.484
2	0.919	0.759	1.114	0.391	0.881	0.703	1.105	0.273
3	0.860	0.704	1.051	0.141	0.970	0.765	1.229	0.800
4	1.150	0.866	1.527	0.334	1.338	0.966	1.854	0.080
5	0.924	0.716	1.191	0.540	0.872	0.650	1.168	0.357
6	1.143	0.776	1.685	0.499	0.967	0.617	1.516	0.884
7	1.062	0.814	1.386	0.657	0.967	0.708	1.322	0.833

Variables with significant *p*-values were selected via logistic regression analysis. OR, odds ratio; CI, confidence interval; number of dates with exercise (0, none; 1, 1 day; 2, 2 days; 3, 3 days; 4, 4 days; 5, 5 days; 6, 6 days; 7, every day); High-Intensity Exercise, 20 min of vigorous exercise; Moderate-Intensity Exercise, 30 min or more of moderate exercise.

**Table 5:** ROC curve analyses for patients with colorectal cancer.

Variable	AUC	Cutoff Value	Sensitivity (%)	Specificity (%)
GGT	0.52	23.50	0.57	0.46
CCI Score	0.58	1.50	0.57	0.55

Variables with significant *p*-values were subtracted using logistic regression analysis. The optimal cutoff points were then pursued via ROC curve analysis. The sex, age, region, and healthcare insurance type were adjusted by PSM (1:1). ROC, receiver operating characteristic; AUC, area under the curve; PSM, propensity score matching (Table S3); GGT, gamma-glutamyl transpeptidase; CCI, Charlson comorbidity index.

## Data Availability

Publicly available datasets were analyzed in this study. These data can be found at https://nhiss.nhis.or.kr/bd/ab/bdaba000eng.do).

## Supplementary Materials

The following supporting information can be downloaded at: https://www.mdpi.com/article/10.3390/curroncol31120553/s1, Table S1: Colorectal cancer codes used in the NHISS DB-originated analysis; Table S2: The details on variables used in this study; Table S3: Propensity score matching test.
