# Supplementary material for: Key Risk Factors, Sex Differences, and the Influence of High-Intensity Exercise on Colorectal Carcinogenesis: A 10-Year Cohort Study Based on 1,120,377 Individuals from the NHISS Data
Source: Curr Oncol. 2024 Nov 25;31(12):7494–510. doi: 10.3390/curroncol31120553 (PMC11674726; doi:10.3390/curroncol31120553)
Supplement: Supplementary file 1 [file curroncol-31-00553-s001.zip › curroncol-3268236-supplementary.pdf]

Table S1. Colorectal cancer codes used in the NHISS DB-originated analysis.

	Code	Classification
**Colorectal cancer**	C19	Malignant neoplasm of rectosigmoid junction
	C20	Malignanat neoplasm of rectum
	C218	Malignant neoplasm of anorectum
	D01.1	Carcinoma in situ of rectosigmoid junction
	D012	Carcinoma in situ of rectum
**Control**	J00	Acute nasopharyngitis

The above colorectal cancer-related codes (found in the Korean Standard Classification of Disease and Cause of Death, http://kssc.kostat.go.kr/ksscNew_web/index.jsp) were used for analysis in this study. The control code used in this study was J00, which represents the common cold.

Table S2. The details on variables used in this study.

Variables	2009~2015			2016~2019		
	Variable name	Code name	Note	Variable name	Code name	Note
Drinking alcohol	Weekly drinking days	Q_DRK_FRQ_V09N	0: none, 1: once, 2: 2 days, 3: 3 days	Number of times you drank alcohol in the past year	Q_DRK_PER	1: per week
						2: per month
			4: 4 days, 5: 5 days, 6: 6 days, 7: everyday			3: per year
						4: not drinking
				How many times a week, a month, a year?	Q_DRK_FRQ	Number of times
Smoking	Smoking status	Q_SMK_YN	1: Does not smoke	Smoking status	Q_SMK_YN	1: Does not smoke
			2: used to smoke but quit now			2: used to smoke but quit now
			3: still smoke now			3: still smoke now
Height	Height	G1E_HGHT	cm (Missing values less than 100)	Height	G1E_HGHT	cm (Missing values less than 100)
Weight	Weight	G1E_WGHT	kg (Missing values less than 20)	Weight	G1E_WGHT	kg (Missing values less than 20)
Waist circumference	Waist circumference	G1E_WSTC	cm	Waist circumference	G1E_WSTC	cm
Body mass index	Body mass index	G1E_BMI	Weight (kg) / (height * height) m	Body mass index	G1E_BMI	Weight (kg) / (height * height) m
Systolic blood pressure	Systolic blood pressure	G1E_BP_SYS	mmHg	Systolic blood pressure	G1E_BP_SYS	mmHg
Diastolic blood pressure	Diastolic blood pressure	G1E_BP_DIA		Diastolic blood pressure	G1E_BP_DIA	
Urinary protein	Urinary protein	G1E_URN_PROT	1: Negative (－) 2: Slightly positive (±)	Urinary protein	G1E_URN_PROT	1: Negative (－) 2: Slightly positive (±)
			3: Positive (＋1) 4: Positive (＋2)			3: Positive (＋1) 4: Positive (＋2)
			5: Positive (＋3) 6: Positive (＋4)			5: Positive (＋3) 6: Positive (＋4)
Hemoglobin	Hemoglobin	G1E_HGB	g/dL (*'0' value missing processing)	Hemoglobin	G1E_HGB	g/dL(*'0' value missing processing)
Preprandial blood sugar (fasting blood	Preprandial blood sugar (fasting blood	G1E_FBS	mg/dL	Preprandial blood sugar (fasting blood	G1E_FBS	mg/dL

**sugar)**	sugar)			sugar)		
**Total cholesterol**	Total cholesterol	G1E_TOT_CHOL	* '0' value missing processing	Total cholesterol	G1E_TOT_CHOL	* '0' value missing processing
**Triglycerides**	Triglycerides	G1E_TG		Triglycerides	G1E_TG	
**HDL cholesterol**	HDL cholesterol	G1E_HDL		HDL cholesterol	G1E_HDL	
**LDL cholesterol**	LDL cholesterol	G1E_LDL		LDL cholesterol	G1E_LDL	
**Serum creatinine**	Serum creatinine	G1E_CRTN		Serum creatinine	G1E_CRTN	
**(serum GOT) AST**	(serum GOT) AST	G1E_SGOT	U/L	(serum GOT) AST	G1E_SGOT	U/L
**(serum GPT) ALT**	(serum GPT) ALT	G1E_SGPT	* '0' value missing processing	(serum GPT) ALT	G1E_SGPT	* '0' value missing processing
**gamma GTP**	gamma GTP	G1E_GGT		gamma GTP	G1E_GGT	
**exercise**	1 week_20 minutes or more of vigorous exercise	Q_PA_VD	0: none, 1: 1 day, 2: 2 days, 3: 3 days	1 week_high-intensity physical activity_day	Q_PA_VD_FRQ	Day
	1 week_30 minutes or more of moderate exercise	Q_PA_MD	4: 4 days, 5: 5 days, 6: 6 days, 7: everyday	1 week_moderate intensity physical activity days	Q_PA_MD_FRQ	Day

Table S3. Propensity score matching test.

Variable	Before PSM						After PSM					
	Colorectal cancer (n = 2,802)		Non colorectal cancer (n = 225,713)		p-value	|SMD|	Colorectal cancer (n = 2,802)		Non colorectal cancer (n = 2,802)		p-value	|SMD|
	N or mean	% or std	N or mean	% or std			N or mean	% or std	N or mean	% or std		
**Age**	63.63	12.42	50.36	14.55	<.0001	0.981	63.63	12.42	63.62	12.43	0.975	0.001
**Sex**												
- Male	1759	62.78	107589	47.67	<.0001	0.307	1759	62.78	1758	62.74	0.978	0.001
-Female	1043	37.22	118124	52.33		0.307	1043	37.22	1044	37.26		0.001
**Region**												
-Seoul	475	16.95	42161	18.68	<.0001	0.045	475	16.95	477	17.02	1.000	0.002
-Pusan	198	7.07	16290	7.22		0.006	198	7.07	199	7.1		0.001
-Daegu	125	4.46	11028	4.89		0.020	125	4.46	130	4.64		0.009
-Incheon	133	4.75	12308	5.45		0.032	133	4.75	131	4.68		0.003

-Gwangju	75	2.68	6350	2.81		0.008	75	2.68	75	2.68		0.000
-Daejeon	101	3.6	6759	2.99		0.034	101	3.6	104	3.71		0.006
-Ulsan	44	1.57	5451	2.42		0.061	44	1.57	34	1.21		0.031
-Sejong	4	0.14	506	0.22		0.019	4	0.14	1	0.04		0.033
-Gyeonggi	554	19.77	52182	23.12		0.082	554	19.77	558	19.91		0.004
-Gangwon	108	3.85	6771	3		0.047	108	3.85	109	3.89		0.002
-Chungcheong-do	120	4.28	7657	3.39		0.046	120	4.28	120	4.28		0.000
-Chungcheongnam-do	161	5.75	10527	4.66		0.049	161	5.75	164	5.85		0.004
-Cheollabuk-do	144	5.14	8822	3.91		0.059	144	5.14	142	5.07		0.003
-Cheollanam-do	141	5.03	8975	3.98		0.051	141	5.03	142	5.07		0.002
-Gyeongsangbuk-do	194	6.92	12709	5.63		0.053	194	6.92	192	6.85		0.003
-Gyeongsangnam-do	186	6.64	14992	6.64		0.000	186	6.64	187	6.67		0.001
-Jeju-do	39	1.39	2225	0.99		0.037	39	1.39	37	1.32		0.006
**Income**												
-Medicaid & NHI self-employed/employee subscriber low	471	16.81	34736	15.39	<.0001	0.039	471	16.81	471	16.81	0.999	0.000

-NHI self - employed subscriber Medium	376	13.42	24069	10.66	0.085	376	13.42	377	13.45	0.001
-NHI self-employed subscriber	345	12.31	24270	10.75	0.049	345	12.31	339	12.1	0.006
-NHI employee subscriber Medium	769	27.44	78444	34.75	0.158	769	27.44	769	27.44	0.000
-NHI employee subscriber High	841	30.01	64194	28.44	0.035	841	30.01	846	30.19	0.004

NHI, National Health Insurance; SMD, Standardized Mean Difference; If |SMD| is less than 0.1, it is considered balanced.
